# ‘I do want to ask, but I can’t speak’: a qualitative study of ethnic minority women’s experiences of communicating with primary health care professionals in remote, rural Vietnam

**DOI:** 10.1186/s12939-017-0687-7

**Published:** 2017-10-30

**Authors:** Shannon McKinn, Linh Thuy Duong, Kirsty Foster, Kirsten McCaffery

**Affiliations:** 1 Sydney School of Public Health, Edward Ford Building (A27), The University of Sydney, Sydney, NSW 2008 Australia; 2 Faculty of Nursing and Midwifery, Hanoi Medical University, 1 Ton That Tung, Dong Da, Hanoi, Vietnam; 3 Office for Global Health, Sydney Medical School, Edward Ford Building (A27), The University of Sydney, Sydney, NSW 2008 Australia; 4 Kolling Institute at Northern Clinical School, Sydney Medical School, Royal North Shore Hospital, St Leonard, NSW 2065 Australia; 5 Centre for Medical Psychology & Evidence-based Decision-making (CeMPED), The University of Sydney, Sydney, NSW Australia

**Keywords:** Communication, Ethnic groups, Minority groups, Female, Pregnancy, Vietnam, Maternal health, Qualitative research, Primary health care

## Abstract

**Background:**

Ethnic minority groups in Vietnam experience economic, social and health inequalities. There are significant disparities in health service utilisation, and cultural, interpersonal and communication barriers impact on quality of care. Eighty per cent of the population of Dien Bien Province belongs to an ethnic minority group, and poor communication between health professionals and ethnic minority women in the maternal health context is a concern for health officials and community leaders. This study explores how ethnic minority women experience communication with primary care health professionals in the maternal and child health setting, with an overall aim to develop strategies to improve health professionals’ communication with ethnic minority communities.

**Methods:**

We used a qualitative focused ethnographic approach and conducted focus group discussions with 37 Thai and Hmong ethnic minority women (currently pregnant or mothers of children under five) in Dien Bien Province. We conducted a thematic analysis.

**Results:**

Ethnic minority women generally reported that health professionals delivered health information in a didactic, one-way style, and there was a reliance on written information (Maternal and Child Health handbook) in place of interpersonal communication. The health information they receive (both verbal and written) was often non-specific, and not context-adjusted for their personal circumstances. Women were therefore required to take a more active role in interpersonal interactions in order to meet their own specific information needs, but they are then faced with other challenges including language and gender differences with health professionals, time constraints, and a reluctance to ask questions. These factors resulted in women interpreting health information in diverse ways, which in turn appeared to impact their health behaviours.

**Conclusions:**

Fostering two-way communication and patient-centred attitudes among health professionals could help to improve their communication with ethnic minority women. Communication training for health professionals could be included along with the nationwide implementation of written information to improve communication.

## Background

Vietnam has made noteworthy health advances over the last 25 years, particularly in regards to improving maternal and child health [1]. However, despite this national success story, regional and ethnic health inequalities persist [1, 2]. Ethnic minority groups have been found to be at increased risk of neonatal mortality, stillbirth, childhood malnutrition and stunting [3] and inequalities may be increasing in some areas, such as service utilisation [2]. There are 54 officially recognised ethnic groups in Vietnam, with the largest group, the Kinh, making up approximately 86% of the population [4]. Vietnam’s 53 ethnic minority groups, with the exception of the Hoa (Chinese), are more likely to be poor and living in remote areas than the Kinh majority [3]. While ethnic minority groups are considered to be a national treasure, demonstrating the rich cultural diversity of Vietnam, historically they have been the target of government reforms aimed at improving living standards while largely sidelining traditional culture [3, 5]. Government policy has referred to ethnic minority groups as under-developed and backwards, while depicting the Kinh majority as more socially and economically advanced [6]. These policies have advocated for ethnic minority groups to alter their lifestyles, as their traditional practices are seen as contributing to poverty and disease [6].

Dien Bien Province (DBP) is a small, mountainous border province located in the northwest of Vietnam with a population of approximately 540,000 [7], around 80% of who belong to an ethnic minority group [8]. The population of DBP experiences poverty, and child and maternal mortality at rates much higher than national averages [1, 9–11]. Previous research into ethnic minority health in Vietnam has shown significant disparities in service utilisation, with ethnic minority women less likely to access antenatal care (ANC) and give birth at a health facility [1, 2, 12–14], and ethnic minority parents less likely to seek medical care for their children when they are ill [15]. While geographical and physical access factors such as remoteness, lack of transportation, and difficult terrain are contributing factors to ethnic inequalities in service utilisation [12, 16, 17], it has been argued that ethnic inequalities are also the result of low levels of investment in physical and human capital [18]. Those investments that do exist, such as cash subsidies on housing construction, agricultural grants, interest-free loans [19], and a targeted poverty reduction policy [3] may suffer from low returns due to social discrimination, cultural difference and inadequate information, further driving inequality [18]. Prior studies have shown ethnic minority people experience cultural and interpersonal barriers when accessing services, such as discrimination, poor attitudes from health staff and a lack of culturally sensitive services [4, 8, 16, 20].

The cultural, interpersonal and spatial factors described above are obstacles that may adversely impact the patient-health professional interaction, an essential pillar of primary care. Moreover, with the high level of poverty, lower level of educational attainment, and lack of Vietnamese language and functional literacy skills among many ethnic minority women in DBP [8], it is reasonable to assume the level of health literacy in the population is low [21–23]. Although there is little research on health literacy in low and middle income countries (LMIC), previous research has established an association between low health literacy and experiencing communication difficulties with health professionals [24–26], and experiencing less patient-centred communication [27]. Several studies in other Asian countries with traditionally hierarchical social structures have also found that these power dynamics can flow into the patient-health professional relationship [28–31].

Maternal and child health is a concern for the DBP Provincial Health Service, which has collaborated with the University of Sydney and the Vietnamese Women’s Union (VWU) to deliver maternal and child health workshops for health professionals and community leaders [32, 33]. During these workshops, limited health literacy and communication between health professionals and women have emerged as major issues impacting on quality of care. Conceptual models of the causal pathway between health literacy and health outcomes have suggested that improving communication (i.e. the patient-provider interaction) may mediate the effect of limited health literacy [34, 35]. This conceptualisation of health literacy provides the overarching framework for this research. The aim of this study is to explore how ethnic minority women experience communication with primary care health professionals in the maternal and child health setting. The overall aim of this research is to develop and support strategies to improve health professionals’ communication with ethnic minority communities in Vietnam.

## Methods

### Study design

This study utilises a qualitative design, and takes a pragmatist theoretical stance [36]. Specifically, this study is a focused ethnography. As in traditional ethnographic research, the focused ethnographic approach allowed us to centre culture while containing our focus to specific research objectives. In focused ethnography, the field of investigation is determined by pre-existing research questions, which are generally problem-focused and context specific [37, 38]. Data collection is not reliant on long-term participant observation, as in traditional ethnography, with an emphasis on “time intensivity” over “time extensivity,” whereby a large amount of data is produced in a shorter amount of time, followed by an intensive data analysis process [39].

### Setting

**Fig. 1:**
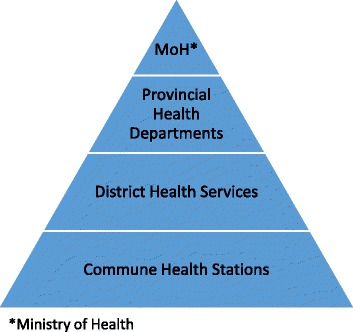
Vietnamese health system structure. *Ministry of Health

We conducted the study in October 2015 in Tuan Giao District, DBP. Tuan Giao district was chosen in collaboration with provincial and district health officials as being a representative rural district at significant distance from the provincial capital (approximately 80 km). The district is divided into 19 communes, with a total population of approximately 82,000 (Son LD, personal communication, Oct 12, 2017). The basic hierarchical structure of the Vietnamese state health system is illustrated in Fig. 1. In Tuan Giao, each commune has a health station, with the District Hospital (which has surgical capacity) serving as the main referral point for all communes. Services at the commune level are staffed by doctors, nurses, midwives (usually responsible for basic maternity care including ANC and normal delivery), medical assistants and pharmacists. Not all commune health stations had a full-time doctor on staff at the time this study was conducted. Although commune level services provide primary care, preventive services, family planning, and maternity care (including normal delivery), in practice, patients often self-refer to district and provincial level services. There is also a small number of private clinics operating in the area.

Most residents of Tuan Giao are from the Thai ethnic minority group [8], with a smaller population of Hmong, Kinh, Khang, and Kho Mu people. Please note that Thai people are a Vietnamese ethnic minority group, as distinct from Thai people who make up the population of Thailand.

### Recruitment

Five communes were selected in cooperation with the District Health Service. These communes were purposively sampled in order to ensure communes with a range of characteristics were included (Table 1).

**Table 1:** Commune characteristics

Commune characteristics	N (%)
Distance from District Hospital (range 4 km – 45 km)	
< 10 km	2 (40%)
10–20 km	1 (20%)
20–30 km	1 (20%)
30–40 km	0 (0%)
> 40 km	1 (20%)
Predominantly sealed road access to District Hospital	4 (80%)
Ethnic makeup	
Predominantly Thai	3 (60%)
Predominantly Hmong	2 (40%)

Women who were currently pregnant, or who had been pregnant in the previous 5 years were eligible to participate in focus groups, and were recruited with the assistance of the VWU at the commune and village level. All participants gave written consent, or gave oral consent after hearing the information in the participant information statement. We provided all participant information and consent forms to participants in Vietnamese, or translated them orally into local languages (Thai and Hmong) if required. All women were compensated 100,000 Vietnamese dong (approximately 4.45 USD at time of data collection) for their time, which the VWU suggested as an appropriate amount. We also conducted semi-structured interviews with health professionals working at the commune health station in each of the five communes; these results are reported separately [40]. Community members were recruited for focus groups without the involvement of health station staff, in order to minimise any perceived or actual coercion. We obtained ethics approval through the University of Sydney Human Research Ethics Committee (Project No. 2015/251), and the research plan was approved and supported by the DBP Public Health Service, the Tuan Giao District Health Service, and the VWU.

### Participants

We conducted seven focus groups with 37 women who were currently pregnant or had children under 5 years old (see Table 2 for participant characteristics) in five villages. Two focus groups were made up of currently pregnant women (PWFG), three focus groups were made up of mothers of children under 5 years (MU5FG), and two focus groups were mixed (MFG). We purposively sampled for diversity, taking into account ethnicity, language spoken, distance of residence from the District Hospital, parity, and degree of health service utilisation.

**Table 2:** Participant characteristics

Participant characteristics	N (%)
Age, years (range 18–33)	
< 20	7 (19)
20–24	21 (57)
25–29	5 (14)
30–34	4 (11)
Ethnicity	
Thai	28 (76)
Hmong	9 (24)
Years of school	
None	5 (14)
1–6	10 (27)
7–12	19 (51)
Post high school	3 (8)
Number of children	
0	9 (24)
1	14 (38)
2	12 (32)
3	1 (3)
4	1 (3)
Currently pregnant	16 (43)

We believe the variation of experience present in the data are sufficient to adequately support the reported results and answer the research questions [41].

### Data collection

Focus group discussions were chosen as the data collection method after discussions with local collaborators. Based on previous experience working with ethnic minority communities in DBP, we felt a group environment, where women could share their experiences and interact with their peers without being outnumbered by “outsider” researchers would be more conducive to an open discussion. Focus groups were made up of between 4 and 8 women, and lasted between 43 min and 1 h and 53 min. We conducted six focus groups in the homes of community leaders, and one in a community hall. Discussions were intentionally held away from commune health stations in order to allow women to speak freely about their experiences with health professionals. Each session was made up of several sections: introduction and consent process (written and/or oral as appropriate), warm-up discussion introducing participants and their babies, focus group discussion (see Appendix 1 for topic guide), and a closing demographic questionnaire. The broad topics covered include 1. Women’s experiences of pregnancy and childbirth, 2. Communication and relationship with maternal health care provider, 3. The role of family and community during pregnancy, childbirth and postnatal period, and 4. Access to and utilisation of health station and services. At several groups, older women who lived in the homes where focus groups were being conducted were also present. Although the older women were not generally present in the room for the entire duration of the focus group, some made comments during the discussion and gave consent to have their contributions recorded by researchers. We also held a focus group for older women; these results will be reported separately. Any men who were present were asked to leave the room for the duration of the discussion, on the assumption that the women could speak more freely about issues around pregnancy and childbirth. Focus groups were conducted primarily in Vietnamese, with some interpretation into Thai and Hmong, and were facilitated by a female Vietnamese researcher with a nursing background (DTL), under the supervision of a female Australian PhD student with extensive experience in qualitative research (SM). Interpretation into local languages was performed by local women, including village representatives of the VWU, the People’s Committee, and in one case a village midwife. The village midwife was not an employee of the commune health station. She was elected by the community to receive village midwife training and received a stipend for her work. We audio recorded all focus group discussions and took detailed field notes, which we discussed in regular meetings throughout the data collection period.

### Data analysis

An independent third party translated audio recording of the focus groups discussions and transcribed them verbatim in English. We used NVivo 11 Software for Windows [42] to manage the transcribed data. We conducted a thematic analysis according to the following steps: 1. One author (SM) reviewed all transcripts and discussed initial impressions with KM and KF who reviewed a subset of transcripts. 2. SM developed a coding framework by coding data using an iterative approach employing both inductive (data-driven) and deductive (researcher-driven) code development. Codes were developed through an initial open coding process, whereby codes were derived from the raw data. Data was also categorised in light of the research aims and questions that guided the development of the focus group discussion guide. Emerging findings from interviews we conducted with health professionals in the same setting were also considered [40]. 3. SM then refined, grouped and categorised codes, comparing codes and examining them across the data set to develop themes. 4. SM summarised themes and discussed them with all authors [43].

## Results

In focus group discussions, Thai and Hmong women on the whole conveyed a wish to learn more about pregnancy, childbirth, their own and their children’s health. They expressed their desire to ask more questions of health professionals. Generally, they wanted more information and access to health professionals, a better understanding of their health and bodies, and more opportunities to participate and learn from health professionals and each other. However, as the following results show, many ethnic minority women were not given adequate opportunity to fulfil these desires, due to the nature of their communication with health professionals. Three main themes emerged regarding how women experience communication with health professionals: 1) the pervasiveness of didactic, one-way delivery of non-specific health information; 2) variation in women’s understanding and subsequent health behaviours and 3) the challenges of interpersonal communication with health professionals. Note that throughout this section quotes marked with an asterisk are in the third person because they are remarks made by local interpreters translating the responses of non-Vietnamese speakers.

### Pervasiveness of didactic, one-way delivery of non-specific health information

Women reported that health professionals delivered information about pregnancy in a didactic, one-way style, with women acting as passive listeners. The information they recalled was mostly general and non-specific in nature, covering areas such as nutrition, check-ups and foetal development, vaccination, general self-care, and taking iron supplements. For example, regarding nutrition, women said health professionals tell them they need to ‘eat enough’, ‘get enough nutrition,’ and ‘eat from all food groups.’ They rarely mentioned being given specific dietary advice, although some women reported they were told they should eat more protein when they were pregnant.

When I had check-ups, they gave me advice. Eat enough nutrition, take proper rest, keep personal hygiene (Thai, PWFG).

They just told me to walk carefully, eat healthy, that’s all. (Thai, PWFG).

The verbal advice given to ethnic minority women by health professionals was supplemented by the Maternal and Child Health (MCH) Handbook. The MCH handbook was discussed in all focus groups, and most women reported receiving one. Women generally reported a lack of explanation of the health information in the MCH handbook from health professionals. Many women, especially the Thai women (where perhaps there is an expectation of higher literacy levels from health professionals than with Hmong women), described being given the MCH handbook, and told to take it home to read, with little or no explanation.

They just gave me the handbook and told me to read it. They didn’t say much. (Thai, MFG).

This may have been adequate for some ethnic minority women, but others stated that they struggled to understand the information in the MCH handbook, both due to the content itself, and the language and literacy barriers.

They didn’t say anything. They just told me to keep it carefully. [laughs] No I don’t [read the MCH handbook at home] (…) Because I cannot read. (Hmong, MU5FG).

Some women particularly specified that they did not have trouble reading the information in Vietnamese, rather it was the information itself they did not understand, while others were unable to read the MCH handbook at all.

They said they do look through the handbook at home but they cannot read so they don’t understand much of it. (…) Some of them cannot read, others can read but don’t understand the information, so they would ask other people around them. (Hmong, MFG)*.

Women frequently reported asking their husband to read the book for them and pass on the information if they were unable to read. Additionally, some women lacked the time or inclination to read the MCH handbook.

They told me to study it at home. There is information (…) everything is in there, it’s just that I was too lazy to read [laughs] (Thai, PWFG).

She doesn’t have time to read it. She works all day, then prepares dinner, then she wants to sleep. (Hmong, MU5FG)*.

However, women still valued the MCH handbook, although not always for reasons related to its function as a source of health information during pregnancy. Even when they could not fully understand the contents of the handbook, women acknowledged its importance and mentioned keeping it as a health record and reference, and even as a sentimental item for their child to read in the future.

Everything in this pink handbook is important (…) it’s just that I don’t understand much. (Thai, PWFG).

This handbook is very meaningful (…) when your children can read, they’ll see how much you love them and they’ll love you back. (Hmong, MFG).

### Variation in women’s understanding and subsequent health behaviours

The minimal detail and non-specific nature of health advice that women described being given to them may lead to women understanding and interpreting health information in a variety of ways in practice, as illustrated by the different perceptions and practices women had around taking iron supplements. Most women who discussed iron supplements had similar perceptions as to why they were prescribed, saying they were necessary when you ‘lack blood,’ to prevent future lack of blood, or for their baby’s health. However, their experiences of communicating with health professionals about iron supplements and how to take them were much more varied. Some women reported general, non-specific instructions like ‘take enough iron,’ and take iron when they ‘lack blood’ (although it is unclear how they would assess this themselves). Others recalled specific, correct instructions about how to take iron supplements. However, women were often unaware that iron should be taken consistently, or were confused about dosage. Some reported they were told to read the MCH handbook for instructions about how to take iron supplements, saying ‘they [health professionals] don’t explain much.’ Women reported inconsistencies between what they remembered being told by health professionals, and what they understood from their MCH handbooks.

They told me to take one pill in the evening. In the handbook, it is suggested to take two or three pills when I lack blood. I asked the doctor and they told me that if I did that I would die [laughs]. (Thai, PWFG).

Several women reported side effects from taking iron supplements. Some women received advice from health professionals to alleviate side effects while others were told they must endure their discomfort as a normal part of pregnancy. Several women reported that they stopped taking iron supplements due to their ‘incompatibility,’ often without telling health professionals. They continued to receive supplements at the health station, although they would not use them. One Hmong woman reported that she began taking her iron supplements again after the village midwife gave her instructions more tailored to her personal preferences.

They told me that there’s no other way, I still have to take the iron for my baby. But I couldn’t. They continued to give me iron but I never took it. I haven’t taken the iron since I started being pregnant. I had constipation. It hurt so much. I couldn’t sit or walk. (Thai, MU5FG).

They told me to take the iron twice a day, each time one pill. But I didn’t take it because I didn’t like the smell. Then [village midwife] came and told me to take just one pill per day, and if I feel nauseous I should take it before sleep at night. (...) Yes I did [take the iron after that]. (Hmong, MU5FG).

### The challenges of interpersonal communication with health professionals

Women reported a range of experiences communicating directly with health professionals, and differing levels of ease doing so, which could be influenced by a variety of overlapping factors, including the language spoken by health professionals, health professionals’ gender, women’s literacy skills, and their comfort asking health professionals questions. Women had differing levels of comfort asking questions of health professionals. Hmong women mentioned that while they were comfortable discussing certain topics with male health staff, such as how to care for a sick child, there were other topics that could not be discussed between the genders. These topics were referred to in the group as ‘sensitive issues’ and were centred around women’s bodies (e.g. vaginal birth). This discomfort prevented them from asking questions about childbirth, and discussing safe delivery locations. This gendered communication barrier did not arise in the discussion with the Thai women, although it should be noted that the Thai women who participated had access to numerous female health professionals at their commune health stations.

She has many questions but she cannot ask them because they [health professionals] are male (…) She cannot ask the male staff about those issues so she has to wait till the female staff comes back to work (…) She can ask male staff about how to take care of the baby, but not questions about giving birth (Hmong, MFG)*.

If women had access to health professionals in more informal settings, such as their homes, some preferred to speak to them there, rather than in a formal health setting.

I ask [name] (…) she works at the health station, so if there is anything I don’t understand, I would ask her. [Name] who lives next to my house (…) She answers my questions about anything. I rarely read the handbook, I don’t have time. (Thai, PWFG).

Other women had a general aversion to asking questions of health professionals, even though they said they felt they could ask health professionals questions. They reported they were confident with the language, and they did not feel that health professionals discouraged question-asking. However, they were reluctant or ‘shy’ to ask health professionals about things they did not understand, which adds extra difficulty to a situation where they are required to be proactive.

Yes, I do want to ask but I can’t speak. (…) I can speak Kinh [Vietnamese] okay (…) I’m shy [laughs]. I don’t understand so that’s it. I don’t ask (Thai, PWFG).

This general aversion to question-asking may also be related to perceptions among women that health professionals may be dismissive of their questions and concerns. Several women described going to the health station when they were worried about something, and feeling they were having their concerns dismissed or effectively ignored by health professionals. One Thai woman reported she had bad stomach pain after taking iron supplements, and was worried about how often her baby was kicking her belly, but on telling the doctor her concerns ‘the doctor didn’t say anything.’

The challenges of interpersonal communication with health professionals extended from one-on-one interactions into the community setting. Although community health education was organised and targeted to women, it often appeared to be poorly communicated to women, or held at inconvenient times. Women who worked outside of the home in the fields often left very early in the morning, and sometimes stayed there overnight, and did not know a session had taken place until after the fact.

We didn’t know. When we came home, they said they did a communication session. We don’t know if they invited us or not but they said we weren’t home. (Hmong, MFG).

I have never been invited (Thai, PWFG).

Women who did attend community sessions reported that health professionals ran out of time to answer questions, adding extra barriers for women who wished to learn more. Time was also a barrier to communication during routine visits to the commune health station.

At the end of the session, the health staff said they ran out of time. If I don’t understand something, I could attend the next session or go to the health station to ask health staff there. (Hmong, MFG).

When I go to the health station, the health staff are always busy, there are so many patients, so many people need them. If I ask them, they wouldn’t have time for other people. (Thai, PWFG).

## Discussion

Ethnic minority women in DBP generally expressed an eagerness to learn more about pregnancy and newborn care. The health information they did recall receiving from health professionals was didactically delivered, non-specific, and often poorly tailored to their situations as ethnic minority women. Health professionals can act as facilitators for ethnic minority women’s understanding of health information, but with the pervasiveness of didactic, one-way communication from health professionals in practice, the onus was placed on women to take a more active role in their communication with health professionals in order to meet their information needs. This may not come easily to them due to challenges including gender, language, time constraints, reluctance to ask questions, and a perceived lack of interest or sympathy from health professionals when women raised concerns about their pregnancies. Additionally, there is a growing reliance on giving women written information, in the form of the MCH handbook. These factors resulted in women interpreting information in various ways, which in turn impacted their health behaviours during pregnancy and motherhood.

There has been little previous research focused on patient-provider communication in Vietnam, generally or in a maternal health context, let alone among a predominantly ethnic minority population. There has been some research into patient preferences regarding patient-provider communication in other Asian LMICs, which has found that people have different communication needs and preferences based on local social norms and cultural context (including traditionally hierarchical social structure) [30, 44]. However, these norms do not necessarily mean that patients in these countries are not open to a more patient-centred communication approach [29, 45]. In Vietnam, a study of decision-making preferences among urban women found a desire for active participation when choosing a contraceptive method in consultation with a health professional, with an autonomous or shared decision-making approach preferred. A passive decision-making approach, in which women’s concerns were secondary to the health professional’s opinion, was evaluated very negatively by women. This was found despite the cultural context in Vietnam which traditionally emphasises hierarchic role differentiation and respect for authority figures [46].

Health professionals working in commune health stations were also interviewed for this study [40]. We found that the commune health professionals generally perceived the main purpose of communication being information delivery, rather than an interpersonal interaction. They perceived the effectiveness of their communication as being based on women’s individual capacities to understand health information, rather than actively reflecting on the suitability of information and materials, or on their own communication skills. This is also reflected in these focus group results, as ethnic minority women and health professionals described a situation in which communication is frequently one-way, both in the clinical and community setting, and driven by the agenda of health professionals rather than by women’s needs and preferences. Health professional-driven care has also been found to impact other aspects of maternal health service utilisation. A qualitative study into childbirth practices in the same province as the current study found that health services failed to accommodate local (i.e. ethnic minority) childbirth preferences, and that the low level of service utilisation was partly due to ethnic minority peoples’ rejection of the medicalised, health care professional-centred approach found in public health facilities [47]. Additionally, it should be noted that health professionals working at the commune level may also be marginalised within the health system as they have limited power and autonomy themselves [16].

Both women and health professionals also described a substantial reliance on sending ethnic minority women home with often complex written information (MCH handbook) in order to meet women’s information needs during pregnancy and afterwards. Our results show that ethnic minority women do value the MCH handbook, particularly as a health record. This corroborates previous qualitative findings from Cambodia which found women value the MCH handbook as a health record and information source, wish to keep it as a reference, and often share it with their family members [48]. However, our findings also demonstrate that often women cannot understand the information inside the MCH handbook, both the content and the language used. Our results indicate that the MCH handbook may be increasing rather than reducing demands placed on ethnic minority women by health professionals by being neither sufficiently understandable (people of diverse backgrounds and varying levels of health literacy can process and understand key messages) nor actionable (people of diverse backgrounds and varying levels of health literacy can identify what they can do based on the information presented) [49]. This is consistent with research in high-income countries which has demonstrated that most patient education materials are too complex for patients with limited health literacy [49].

Previous research on the implementation of MCH handbooks in other LMICs has shown success in increasing ANC attendance [48, 50–52], increasing rates of delivery with a skilled birth attendant and facility-based deliveries [48], improving maternal health-seeking behaviour [53], and in increasing knowledge in specific areas about pregnancy and child health. However, previous research has specified that the MCH handbooks have likely worked to improve these indicators through enhancing communication between health professionals and pregnant women and allowing more personalised guidance to take place. Results from a study in Palestine showed that less-educated women rarely read the handbook at home, but they still became more familiar with health information in the MCH handbook through personalised guidance provided by health professionals who used the MCH handbook [53]. Our findings from DBP show that the MCH handbook is not being used to enhance communication. Instead it is often used in place of personalised and context-adjusted guidance from health professionals, with women being directed to read the handbook at home with little further explanation or opportunities to ask question of health professionals. This passive style of information delivery has previously been found to be a major barrier to health promotion activities among ethnic minority groups in Vietnam, with communication and promotion methods found to be almost entirely passive and information-based, as well as context unadjusted across ethnic groups [54]. Traditionally, formal communication structures in Vietnam have relied on a top-down, one-way hierarchical structure, which has resulted in differences between health knowledge and actual or reported health practices, with high levels of health knowledge not translating into behaviour change. These differences have been found to be due to factors including the use of top-down didactic communication styles, and improper audience segmentation, resulting in inappropriate context-unadjusted messaging and exclusion of specific groups [55]. A recent intervention to improve hypertension control has seen some success in challenging this status quo, showing the acceptability of a culturally adapted storytelling communication approach in rural Vietnamese communities. The storytelling approach was more successful in increasing hypertension medication adherence than didactic content delivery [56].

The MCH handbook used in DBP was piloted in four Vietnamese provinces (of which DBP was one) between 2011 and 2014. The MCH handbook has been evaluated qualitatively and in a pre-post study [57, 58], but almost entirely from the perspective of its usefulness for health professionals and not from the perspective of pregnant women and mothers. One study [57] reported on the prevalence, fragmented implementation and amount of overlap in various MCH home-based records (HBRs) being used throughout Vietnam, and attempted to identify health professionals’ and women’s perceptions of using HBRs, including the MCH handbook utilised in DBP. The reported qualitative results of the study mainly discussed the user experience of health professionals, and only focused on women’s preference to have HBRs integrated into one document - the MCH currently in use in DBP. Another study aimed to assess the MCH handbook in terms of changes in knowledge, attitudes and practices, and also included a qualitative element. While the pre-post study found an improvement in knowledge, attitudes and practices in maternal and child health, the reported qualitative results give little information about how women used and understood the information in their MCH handbooks, or how health professionals used the MCH handbooks as a communication tool [58].

Strengths of this study include a heterogeneous sample, a rigorous analysis process, and the involvement of local collaborators. The main limitations of this study are that Vietnamese is not the first language of the ethnic minority women living in this community, although it is the sole official language of Vietnam. Most women who participated in the study spoke Vietnamese, some with varying levels of confidence, and others needed to speak through local interpreters. However, as this study aimed to capture a wide range of experiences and opinions within the ethnic minority population, we felt it was inappropriate to exclude these women. The use of local interpreters may have also resulted in some distortions in women’s responses, either self-imposed or interpreter-imposed. Local interpreters were often women of high status and influence in their villages (representatives of the VWU, village midwife, People’s Committee employee), and as such women may have censored their own responses, or had their responses altered in translation. This is a cross-cultural study, and as such, some responses may have been misinterpreted by the authors. We have attempted to limit misinterpretations by conducting an independent translation of all audio data, and collaborating with a Vietnamese co-author. The data collection process and any actual or potential misunderstandings were also regularly discussed by the authors in regular meetings during data collection. Additionally, self-reported practice in focus groups may differ from actual behaviour, and there may be a related element of social desirability bias. We have tried to minimise this through the use of a neutral facilitator, and reassuring participants of the confidential nature of their participation. Furthermore, due to the nature of the qualitative approach, the generalisability of these findings may be limited. We have attempted to enhance transferability by thoroughly describing the research context and methods, and relating our results to existing evidence so that readers may better determine the relevance of these findings to other settings.

## Conclusion

The MCH handbook piloted in DBP and three other provinces was earmarked by the Vietnamese government in late 2015 to be scaled up as a nationally standardised HBR document [57]. While a nationally standardised HBR will likely be a useful tool for health professionals, with 54 ethnic groups present in Vietnam, ethnic minority women in other provinces are likely to face some of the same challenges Thai and Hmong women in DBP have experienced. With the move to implement the MCH handbook across Vietnam, government officials and health professionals should be aware of the different experiences and perspectives of ethnic minority women in using the MCH handbook. The results of this study show there is much scope for improving interpersonal communication between ethnic minority women and health professionals in the primary care setting in DBP, including fostering two-way communication and patient-centred attitudes among health professionals. There is an opportunity to include communication training for health professionals along with the nationwide implementation of the MCH handbook in order to ensure that the provision of the MCH handbook enhances rather than replaces personalised communication between pregnant women and health professionals.

## Appendix 1: Focus group topic guide

Introduction and Welcome

1. Pregnancy

- Tell us about when you realised that you were pregnant?
  Prompt: What happened, how did you realise, when did you realise?
- When you realised that you were pregnant, what did you do?
  Prompt: How do you take care of yourself when you’re pregnant?
- How do you know how to take care of yourself when you’re pregnant?
  Prompt: Who do you ask for advice?
  Prompt: What kind of things do they tell you?
- How have you been during your pregnancy?
  Prompt: If they mention issues / complications / illness: what did you do?
- [For mothers]: How was your pregnancy?
  Prompt: If they mention issues / complications / illness: what did you do?

2. Childbirth

- If you have had a baby, can you tell us about the birth?
- Where would you like to give birth? / Where did you want to give birth?
  Prompt: Why?
- Where did you / will you give birth?
  Prompt: Why?
- Who was with you when you gave birth?
  Prompt: What did they do?
- Who would you like to have with you/liked to have had with you when you gave birth?
  Prompt: Why?
- Would you have liked anything to have been different when you gave birth?

3. Communication and relationship with maternal health care provider

- Do/did you visit the health station during your pregnancy?
  Prompt: Why/why not?
- What things do/did they do there for you?
  Prompt: What happens when you go to the health station when you’re pregnant?
  Prompt: What are/were you looking for from the health station staff?
- Do you feel like you can ask the health worker questions about your pregnancy / childbirth / your baby?
  Prompt: What information do they give you?
  Prompt: Is the information helpful?

4. Role of family and community

- What happens/happened after you had your baby?
  Prompt: How is your family involved with the baby? (Husband, Mother, MIL, etc).
- Does anyone (apart from a health worker) give you advice about pregnancy and having a baby?
  Prompt: What kind of information?
  Prompt: Is it helpful?
  Prompt: What do you do if this advice is different from the advice that the health worker tells you.
  Prompt: Whose advice about pregnancy and childcare do you most trust?
- Are there things that your family and or community expect you to do while you are pregnant or when your child is born?

5. Health station

- How far away is the health station from where you live?
  Prompt: How do you get there?
  Prompt: Is it difficult to get there when you are pregnant or have a small child?
- Do you know what services the health station offers for pregnant women and mothers of young children?
  Prompt: What are they?
  Prompt: What do you think of these services?
  Prompt: Do you use them?

## Abbreviations

ANC: Antenatal care
DBP: Dien Bien Province
HBR: Home-based record
LMIC: Low and middle-income countries
MCH: Maternal and child health
MU5FG: Mothers of children under five years focus group
PWFG: Pregnant women focus group
USD: US dollar
VWU: Vietnamese Women’s Union
